# Meeting the Needs of Mothers During the Postpartum Period: Using Co-Creation Workshops to Find Technological Solutions

**DOI:** 10.2196/resprot.6831

**Published:** 2017-05-03

**Authors:** Justine Slomian, Patrick Emonts, Lara Vigneron, Alessandro Acconcia, Jean-Yves Reginster, Mina Oumourgh, Olivier Bruyère

**Affiliations:** ^1^ Epidemiology and Health Economics and Support Unit in Epidemiology and Biostatistics Department of Public Health University of Liège Liège Belgium; ^2^ Obstetrics and Gynecology Department of Medicine University of Liège Liège Belgium; ^3^ Wallonia e-health Living Lab The Labs Liège Belgium; ^4^ Bone and Cartilage Metabolism Department of Public Health University of Liège Liège Belgium; ^5^ Epidemiology and Health Economics Department of Public Health University of Liège Liège Belgium

**Keywords:** mothers’ needs, technological solutions, co-creating workshop, co-creation, postpartum needs

## Abstract

**Background:**

The postnatal period is associated with many new needs for mothers.

**Objective:**

The aim of this study was to find technological solutions that meet the needs of mothers during the year following childbirth.

**Methods:**

Two co-creation workshops were undertaken with parents and professionals. The aim of the first workshop was to create a list of all the criteria the proposed solution would have to address to meet the needs of mothers after childbirth. The aim of the second workshop was to create solutions in response to the criteria selected during the first workshop.

**Results:**

Parents and health professionals want solutions that include empathy (ie, to help fight against the feelings of abnormality and loneliness), that help mothers in daily life, that are personalized and adapted to different situations, that are educational, and that assures some continuity in their contact with health professionals. In practice, we found that parents and professionals think the solution should be accessible to everyone and available at all times. To address these criteria, technology experts proposed different solutions, such as a forum dedicated to the postpartum period that is supervised by professionals, a centralized website, a system of videoconferencing, an online exchange group, a “gift voucher” system, a virtual reality app, or a companion robot.

**Conclusions:**

The human component seems to be very important during the postnatal period. Nevertheless, technology could be a great ally in helping mothers during the postpartum period. Technology can help reliably inform parents and may also give them the right tools to find supportive people. However, these technologies should be tested in clinical trials.

## Introduction

Pregnancy and childbirth are two critical stages in a woman’s life. The postnatal period is associated with many new needs for mothers, and several studies have demonstrated a great need for information after childbirth [1,2]. Many mothers search for reliable and realistic information and want to be better prepared for the realities of motherhood (especially women having their first baby) [3,4]. Many also have anxieties and fears around early parenting and their changing roles [5]. Women are generally concerned about the safety of their new baby, and they lack self-confidence as new mothers and in their own ability to care for their baby. Women need to be surrounded by those who will emotionally support them in this transition to parenthood [6,7].

A previous unpublished study in our department (Department of Public Health, Epidemiology and Health Economics, Liège, Belgium) has evaluated the needs of mothers in the year after childbirth and listed them in four categories: (1) a need for information (women seemed to require medical, practical, and administrative information); (2) a need for psychological support (women want to be surrounded, reassured, and understood in this difficult period of life); (3) a need to share experiences (women liked having the possibility of discussing issues with other mothers, especially to find out if what they are experiencing is normal); and (4) a need for practical and material support (women remained preoccupied by housework and appreciated help with household chores, ironing, etc).

Today, the Internet and new technologies are a constant feature in daily life [8]. For example, in 2015, 75.0% of Belgian people (vs 61.7% in 2014) said that they used the Internet at home to get information [9]. Be it for private or professional purposes, connecting to the Internet to communicate or seek information is now part of our daily life. Innovations in mobile and electronic health care are revolutionizing the involvement of both patients and doctors in the modern health care system, creating new opportunities for patients to participate actively in monitoring and improving their own health, and for doctors to supervise their patients’ health. During the perinatal period, (future) mothers are turning more frequently to the Internet to satisfy their need for information [10,11] but also to help them make decisions [12-14]. In addition, studies have already demonstrated the effectiveness of interventions based on new technologies during the postpartum period (eg, an Internet-based intervention enhancing Finnish parents’ parenting satisfaction and parenting self-efficacy [15], telemedicine after early postnatal discharge [16], and videoconferencing as a support in early discharge after childbirth [17]). Therefore, following a previous exploration of mothers’ needs during the postpartum period, the aim of this study was to find one or more adapted technological solutions to meet the needs of mothers during the year following childbirth.

## Methods

To find technological solutions that meet mothers’ needs after childbirth, two co-creation workshops were organized. The study was approved by the Comité d’Ethique Hospitalo-Facultaire Universitaire de Liège, Belgium (#2015/48).

### Step 1: Make a List of Criteria for Proposed Solutions

The aim of the first co-created workshop was to bring parents and health professionals together to list criteria that proposed solutions must meet to address the needs of mothers during the year following childbirth. We chose to focus on the year following childbirth as similar to the perinatal period, which is defined as from conception to 1 year after birth [18,19]. In addition, postnatal depression is common during this period. Its incidence does not necessarily decline over the first year following childbirth, and it is associated with physical symptoms, especially tiredness or even exhaustion [20,21].

Our inclusion criteria were women or men, who had a child under 2 years and who agreed to participate in the study; and any professionals involved in the postnatal period (ie, gynecologists, midwives, pediatricians, general practitioners, psychologists, medical-social workers of the Office de la Naissance et de l’Enfance [Belgian Office of Birth and Childhood], and nursery nurses). We chose to include fathers because they are well placed to give information about their wife’s experience. In addition, our previous study, which evaluated the needs of mothers after childbirth, showed that fathers play a central role in the psychological well-being of their partner and that health professionals consider fathers as real partners in care. Exclusion criteria for mothers and fathers were the following: multiple gestation pregnancy, fetal death in utero, very premature childbirth (<34 weeks of gestation), and fetal pathologies. There were no exclusion criteria for professionals.

The recruitment of participants was mainly done through social networks: Facebook and the websites of the Wallonia e-health Living Lab (WeLL) and of the AlterNative (platform for a respected birth). Individuals who had already been in contact with our research team and who matched the inclusion criteria were contacted to participate in these focus groups. Professionals were also contacted based on their specialty. During the first workshop, 12 participants were present: 3 midwives (one of them was also a young mother), 1 gynecologist, 1 psychologist, 1 medical-social worker from Office de la Naissance et de l’Enfance, 5 mothers, and 1 father. Two work groups were formed.

The workshop was held at WeLL on December 16, 2015. The workshop protocols were drafted by the research team in collaboration with a group of experts for the co-created study design at WeLL. First, participants were asked to introduce themselves to each other, and the search topic (including the four needs previously identified) was presented. Participants were given the opportunity to ask questions about the study and their involvement before the session started. Then, two work groups were formed. Three of the four needs previously identified were explored in this first workshop: the need for information, psychological support, and shared experiences.

Exploration of the need for information was done by the two work groups. It consisted of listing all the criteria that participants liked (for the first group) and did not like (for the second group) in the actual dissemination of information system. To do this, participants had to come up with “pros” letters (“I am completely in love with you because …”) for the first group, and “cons” letters (“I do not love you because …”) for the second group.

Exploration of the need for psychological support was made with a mind map with the first group only. The mind map consisted of mapping participants’ thoughts on a large sheet of paper. Participants were asked to think about the terms that mothers had linked with psychological support, namely, “surrounded,” “understood,” and “reassured”. Those three words were written on the sheet at the start. Participants could then write down the first word they thought of when reading one of the three words above. Participants could then bounce off their own words or words written by others.

Finally, the need to share experiences was explored by the Chinese portrait method. The principle of the Chinese portrait method is to respond to the question “If I were …, I would be …”. Participants had to respond to the following questions: “If I were a dish, an exotic pet, a song, an object, a perfume, a town, an actor, I would be …”. After each response, participants had to explain why they chose their response.

The need for practical and material support was not explored because there are very few possible technological solutions to meet this need.

Each stage of the workshop highlighted some criteria required for the development of potential solutions. Some illustrative examples of the co-creative methods used in the first workshop and how we extracted the most important criteria required for the development of potential solutions from each method are presented in Table 1. After each step, participants had to choose four criteria that they found essential for the development of potential solutions. Therefore, each participant had to place four stickers next to their most important criteria: the more important the criteria, the more stickers there were.

**Table 1:** Illustrative examples of the co-creative methods used in the first workshop of the study.

Need	Exploration methods	Examples from the first co-created workshop^a^
Need for information	Pros (I am completely in love with you because …)	… You understand that our demand evolves with the development of our child and you can even anticipate it.
		… You are free and sexy, you attract me and you are available at all times.
		… You allow me to be consistent with myself and you comfort me on the legitimacy of my requests which are common to other mothers.
	Cons (I do not love you because …)	… You are too informative and not enough educational: I am drowning and I feel alone!
		… You’re always arriving at the wrong time; or too early or too late!
		… You’ve maintained the myth of the ideal motherhood for too long and you lack realism, liar! Keep your false advertising for you!
Need for psychological support	Mind map (surrounded)	Listening
		Be helped
		Heat
		Without prejudice
		Presence^a^: if needed^a^; on demand^a^; to be heard
		Non-judgment
		Don’t forget yourself
		Find time for yourself
		To be understood/supported in their own experience
Need to share experience	Chinese portrait	“If I were a dish, I would be Spaghetti or Pizza because they are easy to prepare and shared in community, they require few dishes, they are not expensive ... This helps to lighten the daily life.” “If I were an exotic pet, I would be a penguin because penguins shuffle in circles, staying tightly packed to keep every one of the huddle, warm (comfort, protection, and the weakest in the middle). This could inspire us in the care of mothers.”

^a^The most important criteria required for the development of potential solutions.

### Step 2: Propose Solutions to Meet Participants’ Criteria

The aim of the second co-created workshop was to bring together technology experts (technology professionals and enthusiasts) to devise solutions in response to the different criteria selected during the first workshop. All technology professionals or people with a particular interest in technology who agreed to participate were eligible for this study. There were no exclusion criteria.

The recruitment of participants was mainly done through social networks (Facebook and the WeLL and AlterNative websites) and by word of mouth. The technology experts, who already worked with WeLL, were contacted to participate in the second workshop. The second workshop was composed of 8 technology experts (5 men and 3 women, all parents). Two work groups were then formed.

The second workshop was also held on the WeLL premises on February 22, 2016. The workshop protocols were drafted by the research team in collaboration with WeLL. First, participants were asked to introduce themselves to each other, and two work groups were formed. The research problem (the needs of mothers during the postpartum period) was presented, and participants were given the opportunity to ask questions about the study and their involvement before the session started. The experts discussed and shared their understanding of the context. Then, all the criteria found to be essential for parents and professionals in the previous step were presented to the experts. The experts had to brainstorm many technological solutions for addressing the needs of mothers and to match at least one of the criterion required for the development of potential solutions (=co-creating stage). All solutions using technology, in any manner whatsoever, were welcomed. It was not necessary to have revolutionary ideas but to have, above all, ideas that could meet the criteria previously highlighted. Finally, role-playing exercises (situations that mothers may encounter in the postpartum period) were presented to the experts, who then explained how the solutions that they brainstormed could help mothers in those situations. The role-playing exercises are presented in Table 2.

### Analyses

With the agreement of the participants, the two workshops were audio-recorded using a Dictaphone and then transcribed verbatim. To ensure confidentiality, all information allowing identification was removed from the transcripts. Management of the data and analyses were made manually. Transcripts were systematically coded by topic and classified into groups of similar issues. To help identify the different themes, handwritten notes were made during the workshops and analyzed afterwards. The thematic content analysis used the analytical method of thematic framework (developed by the National Centre for Social Research [22]). This systematic data extraction created a thematic network, which illustrated the relationship between the themes addressed. In the results section, some direct quotes, which were extracted from the workshops, are provided to illustrate each theme.

## Results

**Figure 1:**
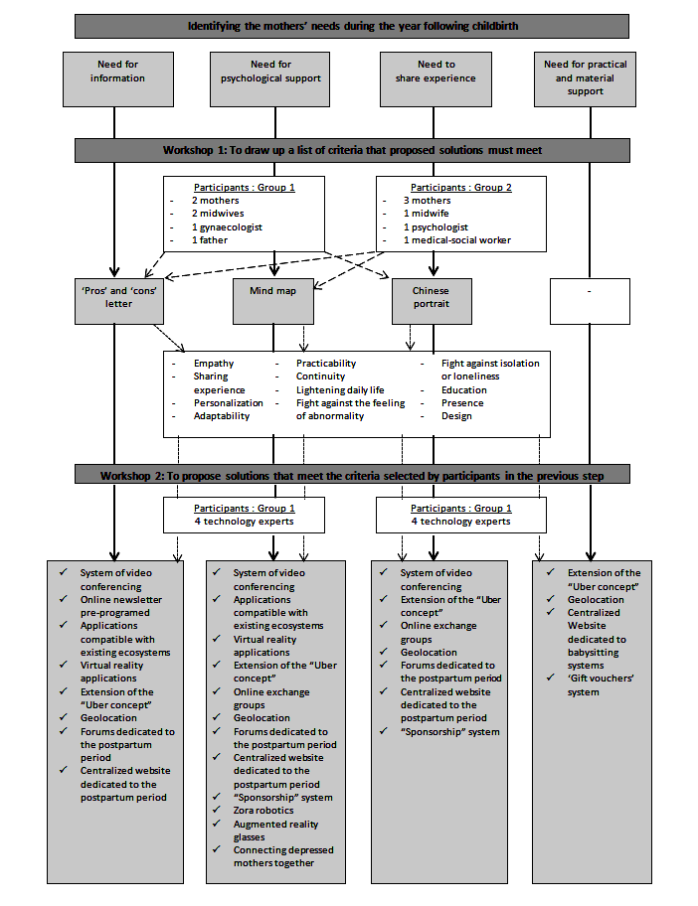
Diagram of the progression from the workshop 1 comments to the workshop 2 solutions.

A diagram illustrating the progression from the first workshop comments to the second workshop solutions is presented in Figure 1.

### Step 1: Make a List of Criteria for Proposed Solutions

The criteria that participants selected during the first workshop are presented in Table 3. The number of stickers assigned to the different criteria is also presented (shown as bullets). The number of stickers demonstrated the importance of the criteria for parents and professionals: the more a criterion had stickers, the more it was considered important.

In addition, several times during the first workshop, parents and professionals insisted on the fact that the fathers’ involvement was beneficial to the development of solutions because they also experience the important transition to fatherhood.

**Table 2:** Role-playing exercises used during the second workshop.

Scenario	Case		
	1: Lea, 22 years old, living with her partner, gave birth to her first child 3 weeks ago. She is a nurse in a nursing home and does a lot of sport.	2: Marie, 34 years old, is married and has 2 children including one of 6 years. She gave birth 6 weeks ago. She works as an employee in a pharmaceutical company. She feels alone during her maternity leave.	3: Melissa, 30 years old, living with her partner, gave birth 2.5 months ago. She works as a waitress in a restaurant chain.
1	She would like to share her experience to see if other women are in the same situation and see if what she is experiencing is normal.		
2	She needs information about breastfeeding because her baby does not drink enough.		
3	She feels overwhelmed by all the tasks of daily life and she is no longer able to take some time for herself.		
4	She feels that her husband is not paying enough attention to her and that he is not invested enough since the birth of the baby.		
5	She finds that the opinions of the different professionals she consults are different (sometimes even opposed). She tries to apply exactly everything they said (changing at each consultation) and therefore, she feels completely lost.		
6	She must start working in 3 weeks but has not found a childcare system yet. She is panicking about the idea of leaving her baby to someone.		
7	Her gynecologist advised her to do postnatal physiotherapy, but she does not know any physiotherapist in this field. Her friends have no children yet and do not know how to help her.		

**Table 3:** List of criteria that proposed solution must meet.

Criteria		Importance
**Empathy**		
	Be accepted without judgement in distress	●●●●●●●● (8)
	Empathy, dramatization, lightness	●●●●●●● (7)
	Comfort into “my normality,” “legitimacy of my requests”	●●●●● (5)
	No judgement	● (1)
	Restore confidence in myself	● (1)
	Be reassured	● (1)
	Be understood	● (1)
**Sharing experience / fight against feelings of abnormality**		
	Sharing experiences of mothers	●●●●●● (6)
	Comfort into “my normality,” “legitimacy of my requests”	●●●●● (5)
	Counterpoint to the collective ideal	●● (2)
	Self-mockery	● (1)
**Presence / fight against isolation or loneliness**		
	Presence	●●●● (4)
	To anticipate needs and meet them	●●●● (4)
	Nonintrusive	●● (2)
	Be surrounded	● (1)
	The weakest people in the middle	● (1)
	<3 (= heart, love)	● (1)
**Lightening daily life**		
	Lightening daily life	●●●●●● (6)
	To let go	●●●●●● (6)
	Do not forget yourself	●●●●● (5)
	Loophole, wellness, serenity	●●● (3)
	Inspiring	●● (2)
	A change of scenery	● (1)
**Personalization / adaptability**		
	Costume made	●●●●●●●●● (9)
	Home visits	●●●●● (5)
	We interpret it as we want	●●●● (4)
	Do not forget the father: male perspective too	●●● (3)
	You adapt yourself depending on me	● (1)
**Practicability**		
	Available at all times	●●●●●●● (6)
	Attractive and accessible	●● (2)
	Free	● (1)
	Timeliness of responses	● (1)
	Modern (online) and reliable	● (1)
**Education**		
	If needed	●●●● (4)
	Identify times when a solution is needed	● (1)
	Identification of unexpressed needs	● (1)
	On demand	● (1)
**Continuity**		
	Contact from the beginning (since the beginning of pregnancy)	●● (2)
**Design**		
	Informal format, flexible	●●●●●●● (7)
	With multiple entry points (eg, time route, keywords, experiences)	●●●●● (5)
	Quiet: “we can take the time”	●●●● (4)
	Language adapted at a social level	●●● (3)
	Comprehension and interpretation of the questions	●● (2)
	Scalable request	●● (2)
	Pedagogy decision support, enlightened information	● (1)
	Colored	● (1)

### Step 2: Propose Solutions to Meet Participants’ Criteria

Several times during the second step of this study, the two work groups both underscored the importance of focusing not only on the technological side during the postnatal period but also on the human side.

To fight against isolation, we need someone, we need a presence; something more real than technological.

Technology could help many new mothers ... For example, it would be interesting to have a tracking system to find services or information nearby where you live (eg, research by postal code).

I do not see how we could assist daily life with technology. Technology will only provide information on how to turn to a supportive person; but will always go through a real person. It would be nice, to be able to sound the alarm …

Notwithstanding, many proposed solutions were debated during this step and are discussed as part of the results. These solutions are presented in Table 4.

**Table 4:** Solutions discussed during the second workshop by the technology experts.

Proposed solutions	Explanations of the solutions given by the experts
Videoconferencing system	“Women expressed a great need for information; thus, the goal of this solution would be to provide the most interactive and comprehensive responses. This approach would consist of filming experts speaking on a topic. This solution could provide access to tutorials, testimony, or to a ‘call center’ run by midwives (eg, videophone) and could also then meet the needs of shared experiences and psychological support. Indeed, midwives are able to say, thanks to their great experience with others mothers, if what a mother lives is normal or not.”
Online newsletter preprogrammed	“Some newsletters in paper form that mothers can receive by post already exist. These newsletters provide information corresponding to child development. The experts suggested transforming these ‘paper newsletters’ into ‘IT newsletters’. This concept would lead to a reduction in the cost of paper and provide the opportunity to create alerts corresponding with the baby’s age. The goal of this solution is to anticipate mothers’ questions.”
Apps compatible with existing ecosystems (eg, )	“These applications consist of integrating data from a sensor that could be programmed to respond to the needs of mothers. For example, sensors could be used to study the baby’s sleep quality, the temperature of the baby’s room or the walk of the mother. Warning messages may also be sent when a mother walks too much or too little. Information messages could also be transmitted directly to the mother to reassure her and to decrease the level of stress caused by the arrival of a child.”
Virtual reality apps (eg, serious gaming)	“The experts suggested establishing some virtual scenarios to prepare mothers to learn how to become a mother (eg, deal virtually with life situations with a baby). Nevertheless, they felt that it was quite difficult to implement this solution because it would suggest an evaluation of mothers, and no one was qualified to do so. Mothers might feel judged or would compare themselves with other mothers.”
Extension of the Uber concept	“Uber is an intermediation platform linking users and service providers. This platform allows a request to a specialist when needed. The experts in our study believed that such a platform could connect mothers with midwives, physiotherapists, osteopaths, housekeepers, babysitters, etc.”
Online exchange groups (eg, Weightwatchers and Alcoholics Anonymous)	“Our previous study, evaluating the needs of mothers in the year following childbirth, showed that women are not really satisfied by forums or Facebook groups especially because there are a lot of French or Canadian mothers on these groups who live in a different environment with a different culture. Women were looking for mothers living in their area, who gave birth in the same hospital or who have the same doctor. Therefore, the experts came up with the idea of some online exchange groups where women could find mothers from their neighborhood. These online exchange groups would be created by the hospital or by a health professional to try to bring together mothers by region. With these systems, mothers would know that they are not alone.”
Geolocation	“Finding people close to home was deemed very interesting. Indeed, on the Internet, mothers can find people from all over (from different cities or even different countries). Geolocation would provide the possibility of finding a professional near home or to organize meetings with mothers in one region.” “We lose a crazy amount of time trying to find the right people.” (A technology expert who explained her experience as a mother.)
Forums dedicated to the postpartum period, supervised by professionals	“Our previous study showed that many mothers’ forums already exist and are largely used by a lot of mothers, but they do not find them really reliable. These forums must be supervised by professionals if we want to make sure that the information given is of quality. Such geolocation-associated forums could better inform mothers with infants about meetings, which are often very poorly advertised.”
Centralized website dedicated to the postpartum period	“Our experts imagined a website that could address most of the questions mothers ask themselves (eg, give them information they need such as details about places where they can go with their baby). Such a website could target both mothers and fathers, meet the need for information, and also help parents find the people or professionals they need. The experts imagined that professionals could advise this website to the mothers they care for. They saw this website as intermediary middle for carers or directly for mothers during the postpartum period, but not as a substitute for professionals.”
“Sponsorship” system	“Every mother would have a ‘godmother’ assigned. The principle of this system would be to sponsor each young mother with a more experienced mother from the same city. The two mothers could chat online or meet (based on their desire/situation). Our experts suggested a non-profit association, which would be the first point of contact for every young mother who needs a referral or to talk with someone experienced.”
Centralized website dedicated to babysitting systems	“Finding a babysitting solution induces stress for many mothers. To avoid having to phone each facility and to be registered on all waiting lists, the experts envisioned a website that mothers could visit to see a list of available spots at each day nursery.”
Zora robotics	“Currently designed to support the elderly, such a robot could accompany mothers to fight loneliness and to provide them some psychological support.”
Augmented reality glasses	“Relaxation programs through virtual reality or augmented reality allow the mother to go into states of ‘total de-stress’ (eg, by putting on glasses, the mother finds herself at the sea, mountains, or wherever she wants to go for 20 minutes a day). These glasses can be rented, and this system would help meet the need to escape.”
“Gift vouchers” system	“Although the need for practical and material support was not explored, the idea of a ‘gift voucher’ system was outlined during the workshop. The experts thought about a system that already exists in Canada. This system invites family or friends of the parents to offer them some help with housework (eg, ironing, cooking, household chores). For example, a friend could offer two hours of ironing to the new mothers instead of a new cuddly toy.”
Miscellaneous	The experts also spoke about a cradle that automatically rocks the baby (which already exists) or connecting together mothers, who are living with postnatal depression.

## Discussion

### Principal Findings

The aim of this study was to find technological solutions that would meet the needs of mothers after childbirth. Parents and health professionals want solutions that provide empathy: mothers need people to understand that the postpartum period is a difficult period of life. Women also need solutions that help fight against feelings of abnormality, which many mothers feel: they want to know if other mothers experience similar situations. Fighting loneliness is essential for developing such solutions: many mothers feel alone during the postpartum period and want potential solutions to find resources to help fight this feeling. The ideal solution would help mothers in daily life: they want the solution to help them find some serenity to get through the postpartum period. Solutions should be personalized and adapted to different situations. They also want the solution to be educational and respond to the questions of the mothers. All of these criteria match the need for information, shared experiences, and psychological support already demonstrated [1-4,6,7]. Mothers also seem to need some continuity in their contact with health professionals; they would like to address the same person from the beginning of pregnancy to the end of the postpartum period. This need for continuity has already been shown in several studies [23-26]. In practice, parents and professionals think that the solutions should be accessible to everyone and be available at all times. The participants in the first workshop also proposed that solutions also include fathers. It would be very interesting to do this work again with fathers, but doing so did not meet the objectives of the study.

In regard to these criteria, some technology experts tried to propose potential solutions for helping parents and health professionals. Some of the solutions proposed seem difficult to implement in real life; for example, the virtual reality app or companion robots. Nevertheless, some solutions could meet the needs of mothers during the postpartum period. There were many promising ideas during the second workshop, such as a forum dedicated to the postpartum period supervised by professionals, a system of videoconferencing, an online exchange group, a sponsorship system, or a centralized website dedicated to babysitting systems. The system of videoconferencing already showed some beneficial results in cases of early discharge after childbirth. Indeed, a study [17] demonstrated that using videoconferencing can facilitate a meeting that makes it possible for new parents to be guided by the midwife in their transition into parenthood. In addition, this system was also appreciated by the midwives who were using it [27]. They judged this system easy to handle, useful for making assessments, valuable, and functional.

Some solutions could also be bundled together. Indeed, we can imagine a centralized website with many functions. Such a website could contain a forum dedicated to the postpartum period supervised by professionals. Health professionals could give interactive responses to mothers’ questions through a system of videoconferencing. The website could provide the possibility of creating an online exchange group, whereby mothers or parents could meet each other. The exchange group could facilitate the possibility of a mother having a “godmother” with maternity experience, who could become a referent for her. The website could also link to a centralized website dedicated to babysitting networks to reduce the stress induced by the lack of places in day nurseries. An extension of the Uber concept could help mothers find the right people/professionals more easily. A website has already been tested to improve parenting satisfaction and self-efficacy during the postpartum period [15]. Nevertheless, no intervention effects were found: all the parents, having access to the website or not, improved their satisfaction and self-efficacy. The authors concluded that more research is needed in this field.

Another interesting idea is the geolocation system. Indeed, parents seem to be struggling to easily find the people they need. Experts spoke about a geolocation system to find a professional near home or to organize meetings with mothers in the same region. Such a system could also help parents find activities to do with the baby, service vouchers (ironing, housework, etc), institutions, or even shops for children. Some geolocation strategies are already being used for research and public health surveillance and management [28], even reducing urgent response times [29] in some regions. We can imagine using this method not only to meet the needs of the parents during the postpartum period but also in many other situations.

Finally, although the purpose of the workshop was to focus on the technological nature of the proposed solutions, the human component remains important not only for parents and health professionals but also for the technology experts. The transition to motherhood is a potentially vulnerable time for mothers’ mental health [20,21,30,31]. Women have fears and anxieties around early motherhood and their changing role [5]. When mothers understand their babies and are able to respond to their baby’s needs, they experience some feeling of security about their new role as mothers [32]. Mothers consider that the actual presence of professional care providers [5] and the family and friends’ support [32,33] could help them feel more secure during the postpartum period. Indeed, women are more likely to experience postnatal depression or anxiety if they feel they have low social support [34,35]. The postpartum period is therefore a difficult period of life, in which mothers (and fathers) have to be surrounded by support [7], and the human component cannot be ignored: a birth is above all a human experience. However, technology can help parents find reliable information, find the people they need (eg, professionals, godmothers, friends), and also bring them some comfort. For example, Danbjørg et al [36] developed an app for Parents Being Discharged Early Postnatally. They found that through the app, parents felt a sense of comfort, which is essential to start living parenthood positively. Additionally, another study [37] insists that technology is imperative for educating mothers (eg, credible electronic linkages, mobile phone technology, videos and access to provider and hospital websites). This access to information must be guided by care providers. By addressing the needs of mothers, women may be better able to experience parenthood with confidence because they would be better prepared and would *feel* better prepared.

### Strengths and Limitations

This study can potentially add to the knowledge that technological solutions may meet the needs of mothers during the postpartum period. This study explored some original co-creating methods that were drafted and exploited in collaboration with a group of experts for the co-created study design. The methods were therefore rigorous and strong. In addition, the participants came from all fields we wanted to represent within the focus groups.

However, our study also presented some potential biases. First, the sample was composed of voluntary participants, which can limit the extrapolation of the results to all mothers in Belgium. Second, having both parents and professionals together in the first workshop could represent a bias. Indeed, parents might not have felt comfortable raising certain issues or disclosing certain information while health care professionals were in the room. Nevertheless, none of the participants knew each other or even had met before and exchanges between parents and professionals were very relaxed during the whole workshop.

### Conclusions

Although the human and psychological components remain very important in the postpartum period, many interesting technological solutions can be used to address the needs of mothers. Technology could be a great ally for meeting the needs of mothers during the postpartum period. The technology could help to reliably inform parents, boost their security senses, and give them the tools to find the right people. Nevertheless, these technologies must be tested among mothers’ cohorts in clinical trials.

## Abbreviations

WeLL: Wallonia e-health Living Lab
